# Author Correction: Divergent reflections around the photon sphere of a black hole

**DOI:** 10.1038/s41598-021-97272-w

**Published:** 2021-08-30

**Authors:** Albert Sneppen

**Affiliations:** 1 Niels Bohr Institute, University of Copenhagen, Blegdamsvej 17, Ø 2200 Copenhagen, Denmark; 2 Cosmic Dawn Center (DAWN), Copenhagen, Denmark

Correction to: *Scientific Reports* https://doi.org/10.1038/s41598-021-93595-w, published online 09 July 2021

The original version of this Article contained an error in the spelling of the author Albert Sneppen which was incorrectly given as Albert Snepppen.

The original Article has been corrected.

